# Sense and Mind method: an innovative methodological approach to embodied rehabilitation

**DOI:** 10.1007/s10339-025-01299-0

**Published:** 2025-08-28

**Authors:** Annalisa Risoli, Alessandro Antonietti, Laura Colautti, Sara Magenes, Giulia Purpura, Leonardo Fogassi

**Affiliations:** 1 Department of Psychology, Università Cattolica del Sacro Cuore, Milan, Italy; 2 School of Medicine and Surgery, University of Milano Bicocca, Monza, Italy; 3 IRCCS Fondazione Don Carlo Gnocchi, Milan, Italy; 4 Present Address: Department of Medicine and Surgery, University of Parma, Parma, Italy

**Keywords:** Neurorehabilitation, Action, Embodied cognition, Executive functions, Spatial cognition, Mental imagery

## Abstract

As neurorehabilitation research expands, it is crucial to ensure that scientific findings are integrated into neurorehabilitation clinical practice. Building on evidence about embodied cognition, this paper proposes an innovative method called Sense and Mind (SaM), designed for individuals with neurodevelopmental and acquired neurocognitive and neuromotor impairments. It aims to rehabilitate spatial cognition and executive functions from the patient’s bodily experience. A description of the theoretical bases of the SaM method is provided. Theory construct involves neuroscientific evidence relative to embodied cognition, movement and action, spatial representation, mental imagery, and executive functions.

Furthermore, a description of the methodological structure is outlined, allowing for interventions with the patient at different levels of complexity and with various goals through a restitutive approach, ranging from programming voluntary movement to constructing and using mental images. Through different goal-directed activities based on multimodal sensory experiences, the SaM method focuses on recovering executive functions, which are crucial for daily life. The SaM method, through an individualised approach based on each patient’s psychomotor profile, can be helpful for neuromotor and neuropsychological rehabilitation of several types of disabilities. Further studies are necessary to investigate its efficacy on larger samples of patients.

## Introduction

To optimise the rehabilitation processes of patients with complex neuromotor and neuropsychological conditions, researchers and clinicians must collaborate to develop and implement new feasible and evidence-based approaches that translate neuroscience insights into good clinical practices for health professionals (Valero-Cuevas et al. 2024; Winstein et al. 2014; Zou et al. 2025).

In this context, the Sense and Mind (SaM) method was developed; it is a rehabilitative approach primarily targeting individuals with neurodevelopmental and acquired neurocognitive impairments, particularly those involving voluntary movement and executive functions (Risoli 2019; Risoli et al. 2015). It is mainly based on the scientific theories of voluntary behaviour programming (Rizzolatti et al. 1988, 1998; Scott and Kalaska 2021) and embodied cognition (Gallese and Lakoff 2005; Lakoff and Johnson 1980). In particular, this rehabilitation approach takes into consideration neurophysiological data from several studies asserting that cognitive processes are closely connected to the body and the actions performed by the individual (Harbourne and Berger 2019; Kuehn et al. 2018; Sivertsen and Normann 2015). Therefore, the SaM method focuses its structure on the assumption that the physical and mental dimensions are strictly interconnected and act synergistically. Consequently, it aims to intervene systematically and progressively on both the spatial and temporal aspects intrinsic to voluntary motor behaviour programming and its cognitive components (with particular attention to the executive system, which is crucial for goal-directed behaviours) (Lezak 1982).

Through structured bodily experiences, the SaM method promotes the progressive and comprehensive elaboration of mental representations of the body into its physical environment (Di Vita et al. 2016; Sirigu et al. 1991). This is allowed by integrating crucial physical and mental components, such as multisensory integration and spatial, temporal, and rhythmic aspects of bodily movement (Risoli 2019).

This is in line with recent evidence suggesting that not only may we utilise bodily experience to enrich our cognitive repertoire but also that offloading abstract reasoning onto physical actions within a tangible and enjoyable environment enhances learning and promotes long-term retention of information and skills (Zou et al. 2025).

In detail, according to the SaM method, interventions may be tailored to the specific functioning of the patient through protocols characterised by goal-directed activities focused on the own body and somatosensory inputs related to it. Successively, this method aims to have the patients represent in various ways what they have experienced during the initial sensory-motor activities, fostering spatial cognition and executive functions (for more details, see the paragraph “Possible practical applications of the SaM method”). This point plays a fundamental role in processing at a higher level of abstraction what has been experienced through the body, thanks to the exploitation of executive functions, such as inhibition, working memory, action planning and monitoring, and mental imagery.

To achieve these goals, the SaM method adopts a bottom-up modality. First, the intervention on the structure is scheduled (World Health Organization 2001), mainly focusing on integrating the bodily representations at a sensorimotor level. Afterwards, the progressive integration of mental representations is carried out based on the structured and goal-directed experiences the patient has undergone. This appears crucial to enhance voluntary behaviour programming in different spaces (personal, peri-personal and extra-personal). Moreover, the therapist constantly pays attention to the executive functions involved in daily actions (e.g., inhibition, working memory, shifting, and monitoring) and can intervene in the executive strategies the patient employs, both in simple actions and complex activities that require high-level planning abilities.

The intervention approach of the SaM method is mainly restitutive (Robertson 1999), concerning the spatial and temporal issues (e.g., voluntary behaviour programming, exploration of spaces, spatial and temporal memory) that are involved in the mental representation of the body and the related cognitive abilities (such as the executive functions). This approach supports the generalisation of the acquired abilities to enhance performance in everyday life activities, such as writing, navigating open spaces, organising daily duties, and school and work activities.

## Theoretical framework

**Fig. 1:**
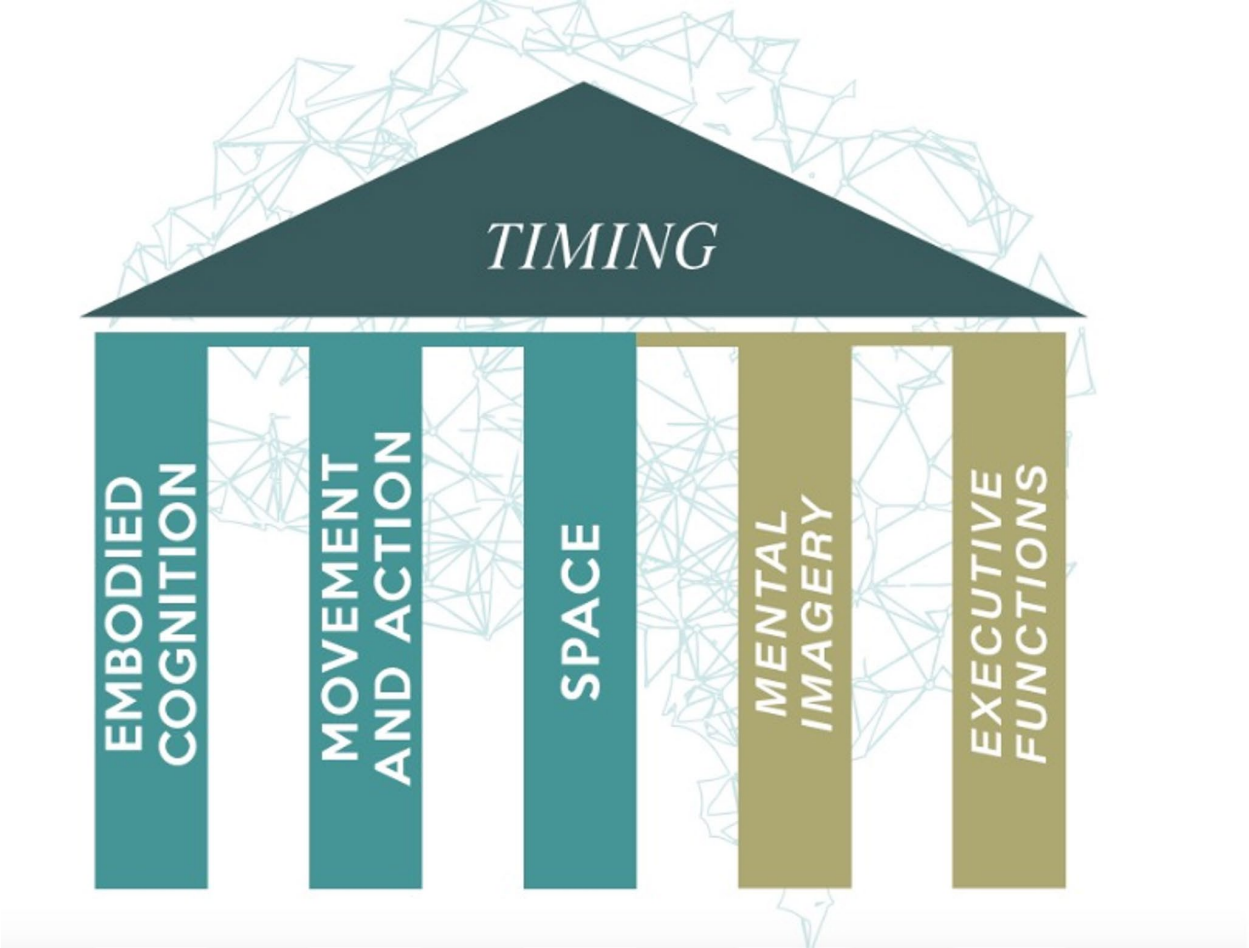
The SaM method’s theoretical evidence

Among the fundamental contributions supporting the SaM method, it is worth mentioning the bio-psycho-social and psychomotor approach by Morosini (Morosini 1978), the “process-oriented” approach by Rahmani (Rahmani 1987), the Terzi Method (Iorio et al. 2015; Terzi 1995), the studies by Montessori and Romagnoli (Regni and Fogassi 2019; Romagnoli 1924). However, the method’s distinctive features are based on neurophysiological evidence, which confirms the importance of bodily experience in all daily activities, according to the theory of embodied cognition (see Sect. 2.1). Unlike other rehabilitative approaches that focus on body function and structures using exclusively neuromuscular facilitation or therapeutic handling, the SaM method primarily considers the active experience of movement, focusing on motor learning processes and exercise that strengthen the executive functions, among the other cognitive aspects involved in voluntary movement, according to most recent systematic reviews and metanalyses (Brusola et al. 2023; Dorsch et al. 2023; Te Velde et al 2022). Thus, the SaM method allows interventions in mental images and executive functions starting from structured bodily experiences (for a synthesis of the method’s theoretical evidence, see Fig. 1) (Risoli 2019).

Before addressing the concept of embodied cognition in more detail, it is also worth clarifying the meanings of the definitions we will use throughout the manuscript to refer to bodily movement organisation and control. We will define “movement” in a strict sense, the displacement of a joint, as “motor act” a movement synergy aimed at a specific motor goal, and finally, as “action” a sequence of motor acts aimed at achieving a given behavioural goal. These distinctions will be detailed below (Sect. “Movement and action”).

###  Embodied cognition

The theoretical framework of embodied cognition postulates that cognitive processes are intimately connected with the body and physical actions. It assumes that (i) the sensorimotor system plays a crucial role in cognitive functioning (Barsalou 2010; Gallese and Lakoff 2005; Lakoff and Johnson 1980; Thelen 1995) and (ii) abstract representations arise from the space of the body (e.g., proprioceptive, tactile, vestibular, and interoceptive ones) (Borghi et al. 2019; Newcombe et al. 2012; Vigliocco et al. 2014; Villani et al. 2021).

According to neurophysiological evidence that considers bodily movement as the primary instrument for knowledge, already from intrauterine life, the relationships between movement and perception play a key role in the individual experience (de Vries et al. 1982; Festante et al. 2018). In fact, from birth, the bodily movement represents a solid developmental scaffolding to sustain human intentionality and social interaction in the environment (Adolph and Hoch 2019; Delafield-Butt et al. 2018). Noteworthy, a preferential tool to assess the nervous system integrity in the perinatal qualitative evaluation is the observation of the infant’s general movements (Cioni et al. 1997; Lüchinger et al. 2008).

### Movement and action

Voluntary behaviour results from complex programming at the cortical level (Rizzolatti et al. 1988, 1998). It is conceptualised as the physical expression of the individual’s intention to reach a goal by acting or modifying the contingent context but also including the choice not to act (Scott and Kalaska 2021). The concept of voluntary behaviour is closely interconnected with the action’s meaning. As mentioned above, the main characteristic of an action is its final goal (e.g., grasping a glass of water and drinking from it). In this sense, the action consists of a sequence of motor acts organised according to a specific order to reach a specific motor goal (e.g., the action of grasping to drink is composed of the acts of reaching, grasping the glass, bringing it to the mouth, opening the mouth, and grasping the glass with the mouth). In turn, each motor act has its own goal (for example, the grasping act has the goal of taking possession of the object), and the implementation of all motor acts in the sequence allows one to achieve the final action goal. Note that this action organisation is built ‘offline’, namely before the individual starts moving. This means that the individual elaborates a motor intention, which corresponds to the internal representation of the final action goal. This organisation also includes the programming of the fluent unfolding of the action, which is possible because every motor act of the sequence is prepared during the execution of the previous motor act. After an action is organised, the various motor acts composing it must be implemented through the execution of the synergy of movements composing each motor act. Finally, these movements can be executed by controlling muscle contraction.

**Fig. 2:**
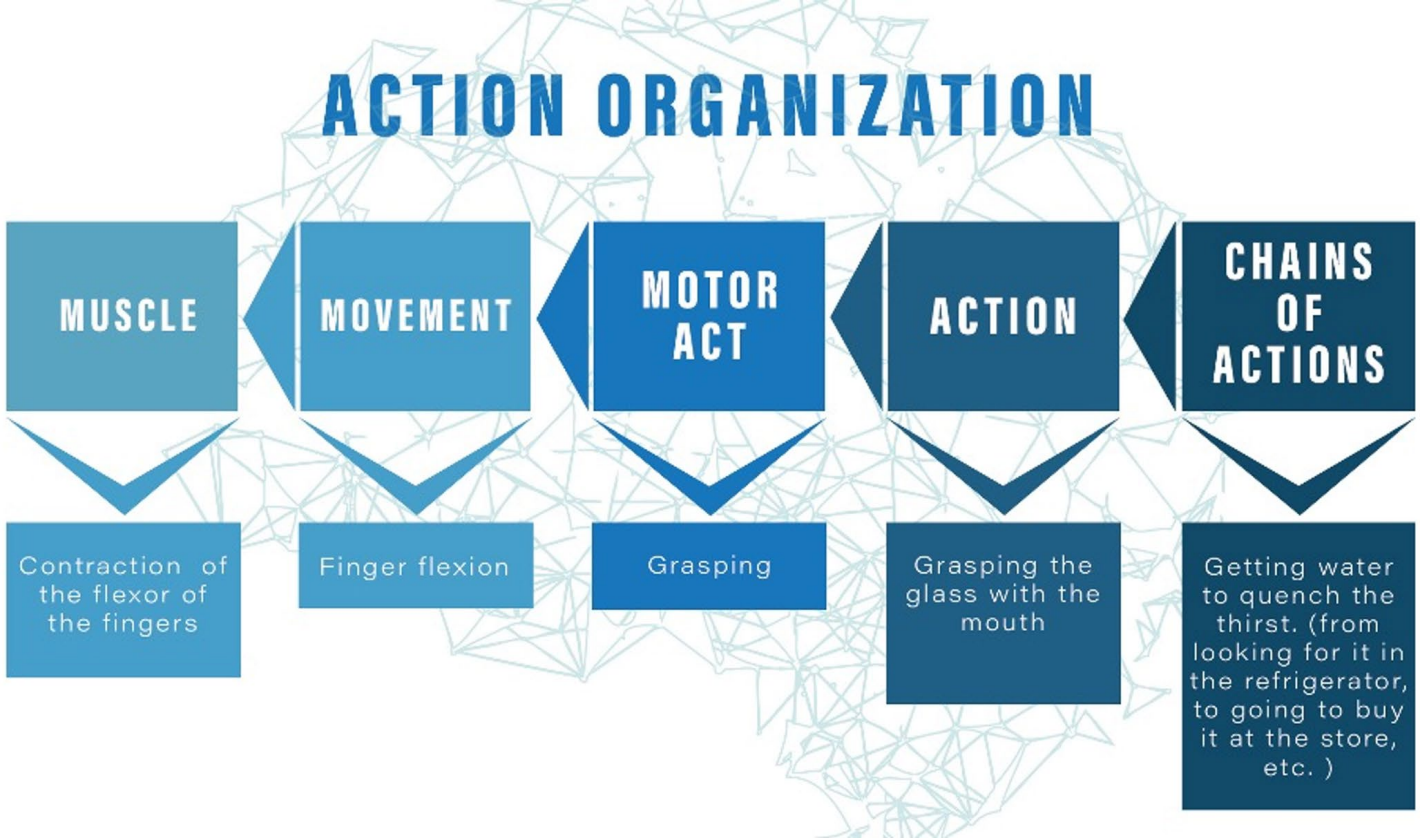
Different hierarchical levels of action organisation and control

Different hierarchical levels of action organisation and control correspond to different anatomical levels. The coding of the final goal occurs at the level of the prefrontal cortex, while the coding of the goals of motor acts involves neurons of the premotor cortex. The primary motor cortex commands the different movements through corticospinal and cortico-brain stem connections. Finally, spinal motor neurons constitute the final output, determining muscle activation (see Fig. 2).

Action organisation necessarily requires mechanisms of sensorimotor transformation performed by several parieto-premotor circuits. These transformations allow individuals to choose the effectors and motor acts suitable to interact with space and objects of the external world. In this way, spatial information is transformed in oculomotor behaviour or reaching acts, depending on the target’s distance from the individual. Similarly, object features (shape, size, orientation, etc.) are transformed into appropriate hand-motor acts. Furthermore, the parieto-premotor circuits mentioned above are controlled by the lateral part of the prefrontal cortex that, based on context and motivation, selects the type of action to be executed or inhibited.

Neurophysiological studies carried out on the parietal and premotor cortex in non-human primates demonstrated the presence of peculiar neurons in different circuits subserving distinct motor-cognitive functions, such as:

- Bimodal neurons of the parieto-premotor circuit for reaching into space that are tactile and visual neurons representing the space around the individual (peripersonal space) in terms of potential reaching or orienting motor acts (Fogassi et al. 1996).
- Visuomotor neurons of the parieto-premotor circuit for grasping that are neurons (“canonical” neurons) that, based on visual object affordances, represent the type of grip to be used for grasping that particular object (object pragmatic description) (Murata et al. 1997).
- Mirror neurons of the parietal-premotor circuit for action recognition that are neurons responding both to the observation of others’ motor acts and the execution of the same motor acts, allowing one to understand others’ behaviour (Gallese et al. 1996; Rozzi et al. 2008).

These three types of neurons share similar mechanisms: they code external visual stimuli (space, object, others’ acts) in a motor format. This confirms the proposal that our primary, first-person knowledge of the world is based on the motor system. These data support the possibility of intervening in cognitive and executive functions through actions in different spaces.

### Spatial representation(s)

Recently, evidence showed that space representation is encoded in many cortical and subcortical regions, and each representation underlies a reference system that involves a specific set of effectors (Hadjidimitrakis et al. 2019).

Space is conceptualised as personal, peripersonal, and extrapersonal:

- *Personal space* involves the space in which our body is settled. Mental representations of personal space result from a complex cognitive process in which proprioceptive and tactile information is integrated with vestibular and interoceptive information (Grechuta et al. 2019; Kilteni and Ehrsson 2017; Raimo et al. 2021). The interaction of this type of information creates a neural “body schema,” which is crucial for selecting behavioural goals and controlling ongoing bodily movement (Scott and Kalaska 2021).
- *Peripersonal space* is the space that can be reached with the arm. It involves reaching, grasping, and manipulating objects and tools (Rizzolatti and Sinigaglia 2007). Peripersonal space representation can be dynamically modified by individual experience, as well as learning allows one to change one’s body (Iriki et al. 1996; Maravita and Iriki 2004).
- *Extrapersonal space* is the space outside the arm reaching distance. An individual can explore this space using oculomotion or walking towards specific targets. In the coding of extrapersonal space, the vestibular system also plays an important role because it continuously provides the individual with inputs about the position and movement of her/his body and head in space, integrated with other sensory information. Within the notion of extrapersonal space, navigational space is also included. This latter is the space in which we move using topographic information based on memorised spatial references and the relations among them. The capacity to form spatial maps depends on peculiar neurons found in the hippocampus and the entorhinal cortex, the place cells and the grid cells, respectively. These neurons allow individuals to form a spatial map that is possible to navigate using the most efficient pathways (Moser et al. 2017; O’Keefe and Nadel 1978).

### Mental imagery (and spatial cognition)

Mental imagery refers to the possibility for an individual to activate internal, sensory or motor representations intentionally (Pearson et al. 2015). It is the conscious generation of an image within a specific domain. If the domain is vision, it is the ability to generate a visual image. If the domain is movement, it is the ability to create a motor image. The generation of mental imagery is therefore made possible by activating mental representations in the areas that constitute the substrate of the imagined domain. So, for visual imagery, mental representations are activated in the visual areas, while for motor imagery, in the motor areas.

The voluntary generation of mental imagery occurs from within (e.g. when the individual consciously decides to imagine a flower or an action). Individuals can also activate mental representations in a specific domain without being aware of such activation (e.g., a passive activation of mental representations occurs, thanks, for example, to neurons with multimodal properties). Alternatively, it is possible to activate these representations in an indirect and implicit way (e.g., when these representations are needed for us to do or decide something).

#### Motor imagery and motor representations

The term “motor imagery” refers to the internal simulation of a bodily movement/action without a real execution (Jeannerod 1994; Jeannerod and Decety 1995). It occurs when it is voluntarily represented by someone doing an action due to the activation of domain-specific motor representations. Specifically, if we imagine ourselves, we can only refer to kinesthesia, while sight is generally not activated. If we imagine another person doing an action, the mirror system is activated internally, and we create a simulation of the action. The presence of motor imagery is typically assessed using the paradigm of mental chronometry (Jeannerod 1994). However, indirect and implicit activation of a motor representation can occur, for example, with the Parsons Hand Laterality Judgment task (Parsons 1994), which requires solving a task in which the subject can implicitly recall a motor representation by performing a mental rotation. Motor imagery has been utilised in rehabilitation for various pathologies (Dickstein and Deutsch 2007; Errante, et al. 2019a, b; Wilson et al. 2016).

#### Visual imagery

“Visual imagery” is another well-known example of imagination (Kosslyn and Shwartz 1977). This is a fundamental tool in many complex cognitive processes (Pearson 2019; Pearson et al. 2015). In accordance with embodied cognition, this is basic for the transition from the concreteness of action to concepts (Gallese and Lakoff 2005) and for the development of spatial cognition (Postma and van der Ham 2016). In fact, according to Kosslyn (Kosslyn et al. 1983), mental images play a role in cognitive processing because they can be used as symbolisation and simulation tools. As symbolic tools, mental images represent objects or concrete events that are replaced by signs. As simulation tools, mental images allow people to form an internal representation that maintains analogical correspondence with the external world. So, visual Images are not ‘photocopies’ of objects or scenes because they do not correspond point-by-point to the original stimulus. Instead, they are mentally organised in structural terms, i.e., by grouping elements in such a way as to form meaningful units. Hence, they enable people to quickly identify the most critical elements from the representation, thereby producing a schematisation in which the essential features are highlighted (Antonietti 1991). In addition, mental images can be analysed and transformed holistically (i.e., by simultaneously acting on all their parts rather than part by part). In this way, they facilitate the processing of information because, by allowing several operations to be carried out simultaneously, they reduce the cognitive load and by permitting people to process information in parallel, i.e., in such a way that various aspects are considered simultaneously, they speed up the process (Kaufmann 1981). The possibility of taking several aspects into account simultaneously is particularly useful when operating in ambiguity and uncertainty, i.e., in situations where it is not advisable to choose one direction straight ahead and exclude the others. However, it is better to consider all possible paths since there are no sure clues as to whether one or the other is preferable (Kaufmann 1988). A further reason is that mental images can be easily manipulated because they are highly flexible representations.

###  Executive functions

Executive functions encompass planning and selecting actions, inhibiting them when they are no longer functional, adapting them flexibly according to abstract rules or contextual and mnemonic information, and monitoring the related processes (Lezak 1982). The role of the prefrontal cortex in daily life actions still has to be investigated in depth because most neurophysiological studies focus on recording neural activity during the execution of tasks based on abstract rules, which often implies the use of short-term memory (working memory). Data from the literature in non-human primates and in humans and the proposed cognitive models suggest the presence, in the prefrontal cortex, of a sort of central control unit (the ‘central executive’) that is formed by two sections, a visuospatial one, with the role of mnemonic retrieval of visual aspects for planning actions in space and a verbal one with the role of mnemonic retrieval and organisation of linguistic material (Baddeley and Hitch 1974). From monkey studies, it is known that the two pathways of ‘what’ and ‘where’, coming from the temporal and parietal cortex, respectively, converge onto the prefrontal cortex thanks to anatomical connections so that this latter can receive information from these two cortical sectors and integrate spatial and semantic information. Indeed, neurophysiological data show that the dorsal part of the prefrontal cortex contains neurons responding to spatially related information, while the ventral part contains neurons responding to objects and faces (for social aims) (Levy and Goldman-Rakic 2000). Thus, the prefrontal cortex receives all types of information, allowing it to select which action to perform in a specific context. In fact, this cortex plays a crucial role in behavioural planning, controlling action sequences, and decision-making.

Even the sequential organisation of language, which is probably derived from action scaffolding, is a typical task of the prefrontal cortex in humans, particularly in relation to speech articulation and the organisation of both simple and complex sentences.

Finally, among executive functions, it is essential to take into account inhibition (Fusi et al. 2022; Colautti et al. 2025). In phylogenesis, inhibition became increasingly important as the evolutionary level became more complex. Humans apply the inhibitory capacity to actions but also to other functions, including moral behaviour. At the motor level, this capacity is demonstrated by disinhibition syndromes, such as patients showing utilisation behaviour, who are not able to, when presented with an object, refrain from reaching and grasping it, even when no instruction to do so is given.

### Temporal aspects

In all our actions, temporal aspects cover a fundamental role and, from a neural point of view, are closely related to space (Buzsáki and Llinás 2017). Temporal aspects are crucial at different levels (e.g., muscles’ activation-deactivation timing, correct sequencing and organisation of motor acts, and basic functioning of the cerebral cortex) (Bruno 2010; Coull et al. 2024; Fraisse 1974). The temporal relationship between events is characterised by succession, duration, and synchrony, in which rhythm represents the basis of bodily movement. The complex temporal organisation of neural activity has also been studied in relation to neuroplasticity and learning (Buzsáki et al. 2013; Hebb 1949; Singer 1993). Researchers who addressed these topics hypothesise a very complex organisation where information is encoded not only in the discharge frequency of neurons but also in the precise timing relations among the discharges (Singer 2021). Temporal sequencing is also fundamental for programming complex actions in which prefrontal lobes are mainly involved.

## The SaM method: fundamentals and applications

### Theoretical model

The SaM method may be considered a manualised psychomotor rehabilitative intervention (Risoli 2019) that, based on the above-described neuroscientific evidence, mainly focuses on:

1. The assumption that the body interacts with the world since the first period of neurodevelopment, shaping cognition and that somatosensory systems play pivotal roles in cognitive functioning (Von Hofsten 2007; Hofsten 2009).
2. The neurophysiological research on sensorimotor learning outlines the action as the initial driving force of knowledge (Gallese and Lakoff 2005).
3. Neurophysiological evidence on programming actions in the parietal and frontal cortex (Errante and Fogassi 2019).

Consistently, voluntary behaviour can be interpreted as a physical manifestation of an individual’s intention to act on the environment to achieve a goal (in the present or in the future) (Scott and Kalaska 2021). Thus, learning can be possible through continuous and higher-level sensorimotor integration. According to the embodied perspective, the somatosensory (proprioception and touch), vestibular, and interoceptive systems play a pivotal role in this process. Furthermore, the central integration of vestibular information is crucial for spatial cognition. In line with these insights, the SaM method involves bodily movement programming through activities that alternate the use of vision and its exclusion to support the integration of proprioception, touch, vestibular signals, and interoception with vision and hearing (Risoli 2019; Risoli et al. 2015).

According to this method, bodily movement is not conceived as the effect of exclusively motor acts but rather in cognitive terms since movement programming necessarily requires spatial information. In fact, space is a basic cognitive function of movement (Rizzolatti and Sinigaglia 2007), and spatial coding is fundamental in all our actions. Accordingly, the SaM method involves the elaboration of different spaces, starting from both the egocentric personal space and the definition of the body axis, as well as the integration of hemispaces (Risoli 2019).

Great importance is given to non-verbal mediation and the creation of a good relationship (Porges 2007), also through the use of the mirror neuron system as a mediator of empathetic relationships between the therapist and the patient (Stern 2010).

Several activities that involve planning and organising voluntary movements in different spaces characterise this method. They have a well-defined structure (for more details, see the paragraph “*The Structure of Activities”*), which allows, through a step-by-step methodology, the fostering of mental imagery, cognitive functioning, and spatial cognition —all fundamental in daily activities. In detail, according to the embodied cognition perspective, mental images play a pivotal role in transitioning from the concreteness of actions to concepts and the development of spatial cognition in the interaction between perception and action. Since early periods of life, spatial cognition develops through spatial memory, which progressively, during childhood and adolescence, enables the generation of mental images and the acquisition of the ability to perform mental rotations and operations on these images.

Due to its complexity, it can be stated that the SaM method integrates two types of approaches to (re) habilitate voluntary bodily movement and its programming, aimed at facilitating the functional adaptation of the individual in daily life. Considering the International Classification of Functioning (ICF) framework (World Health Organization 2001), some activities may be categorised within the body function and structure-oriented approaches because they are designed to improve targeted body functions considered to underlie the functional motor problem of the individual (e.g., body awareness, sensory modulation and integration, active range of movement, tone modulation, balance, attention processes), while some others may be inserted within the activity-oriented approaches, where specific activity is designed to improve specific performance and the content of the intervention involves direct training of the concerned skill (e.g., specific gross-motor activities for personal care, handwriting or other academic tasks, motor imagery, etc…). The integration of these two aspects is a crucial point of this method, highlighting its uniqueness.

###  The structure of activities

The SaM method encompasses two types of activities: Basic Activities, based on errorless learning techniques, and Multimodal Integration Activities, based on sensorimotor experiences that influence cognition, mainly executive functions. Both activities may be carried out in different spaces: personal space (aimed at enhancing mental representations regarding the body schema), peripersonal space (aimed at expanding the repertoire of motor acts, the ability to carry out actions, and plan complex behaviours) and extrapersonal space (aimed at facilitating the exploration of the environment, the planning and execution of the body motion and successively promoting the creation of motor and visual imagery of movement in the environment).

*Basic Activities*, through trial-and-error learning, have a clear and well-defined rhythmic motor structure. They involve the repetition of bodily movements that lead the patient towards the correct movement execution through appropriate perturbations of the motor program, gradually fading through a progressive attenuation process.

*Multimodal Integration Activities*, primarily utilising the reinforcement learning model, encompass various types of experiences, including sensorimotor experiences, that facilitate the integration of implicit and explicit learning through distinct phases of the activity. Indeed, multisensory processes permit combinations of inputs from different sensory systems, allowing for a coherent representation of biological events and facilitating adaptation to the environment (Purpura et al. 2017). Table 1 reports the phases that characterised the structure of the SaM Method.

**Table 1:** The phases of the SaM method

Phase 0	The first phase focuses on the instruction of the activity, which can be motor, but also verbal, visual, or tactile. Visual imitative instruction is often used to activate imitation based learning. Other instructions, such as motor and tactile ones, are often performed blindfolded. The choice of the type of instruction determines the type of sensorimotor integration that is implicitly requested to be performed, and it is essential for Phase 1. For example, in the Multimodal Integration Activity of Point Contacts, the patient, usually blindfolded, is touched with the tip of a finger at certain points of the body, at a constant rhythm (tactile instruction). Alternatively, the patient is guided to perform a circumference, blindfolded, walking in the room (motor instruction).
Phase 1	The bodily experience, always present, stimulates the brain mechanisms underlying motor planning of actions. For example, the patient touches himself at certain points on the body, blindfolded, after having had a tactile instruction in Phase 0. Alternatively, the patient can autonomously execute a circumference by walking, blindfolded, after experiencing the motor instruction in Phase 0. In this way, the sensorimotor experience allows the patient to organize the action in autonomy, retrieving the experience in working memory and organizing the motor behavior to execute the request. In Phase 1, the sensory experience is often performed without sight, to allow the patient to focus on vestibular and somatosensory information.
Phase 2	It is the reproduction of the experience and occurs immediately after Phase 1. After the sensorimotor experience (Phase 1), the patient has to generalize it and reproduce the experience in different ways, using various tools, such as a brush or colors on a paper, if in Phase 1 they performed a circumference while walking blindfolded or replicating the same experience on the operator, if in Phase One individual touched yourself in some places on the body. Through the request for the reproduction of the experience, the intervention is structured on the motor and visuospatial mental images and on executive functions.
Phase 3	It is possible to achieve it through operations on mental images (rotations or flipping) if the subject’s potential development area allows it. Procedures that involve implicit and explicit requests for the rotation of mental images are scheduled. In this way, the passage from first-person to third-person motor images and visuospatial images is supported. As well, the ability to perform operations on mental images is fostered to promote the development of spatial cognition.
Phase 4	The generalization to activities of daily life is encouraged. This phase covers a key role, as in everyday life planning activities can be very complex (e.g., constructing mental maps, timelines, orientation in space, or writing).

The activities are selected and combined within the rehabilitation session according to the patient’s needs, based on a multidimensional and multidisciplinary clinical assessment.

During clinical assessment before and after the intervention, according to evidence-based practice, standardised tools are used to evaluate motor, perceptual, and neuropsychological functions and activities of daily living for the specific disorder and age of the patients. This is necessary to define rehabilitative objectives and verify the effectiveness of the intervention. Moreover, to better plan and monitor the intervention, some selected but non-standardised tasks, inspired by some specific activities of the SaM Method (SaM Evaluation Checklist), are usually administered to the patients.

In this way, the intervention focuses on improving those “building blocks,” which provide the foundation for acquiring or regaining more complex motor, imaginative, social-cognitive, and executive skills.

According to the SaM method structure, caregivers are not directly involved during training sessions, although their role is crucial for some phases of the therapeutic project:

I. Assessment to identify the primary need of the patient: The SaM method provides specific rehabilitation periods aimed at specific functional objectives, which can also be interspersed with other approaches to facilitate the generalisation of learning in different contexts. Thus, the presence of the caregiver is essential to understand the short-term and medium-term objectives and share the therapeutic project.
II. Transfer of abilities in daily life: Based on personalised and tailored therapeutic objectives for each patient, caregivers can adapt specific strategies within daily life activities under the supervision of the therapist. This aspect may encourage patients to utilise their bodies more effectively to understand and explore their surroundings and engage in mental operations by drawing on their bodily experiences.

### Possible clinical applications of the SaM method

Neuroscience evidence on voluntary movement programming and executive functions shows that patients with congenital or acquired disabilities may display difficulties in the egocentric representation of the body and its actions in different spaces due to problems in sensorimotor integration and movement programming, as well as due to impairments in (spatial) executive functions.

Based on these points, the framework of the SaM method has evolved in the field of individual rehabilitation projects, aiming to acquire or regain autonomy in daily life activities in line with the International Classification of Functioning, Disability and Health (ICF) framework (World Health Organization 2001). For this reason, it is implemented by rehabilitation professionals with a qualifying degree (e.g., physical therapists, neuro and psychomotor therapists for developmental age, occupational therapists, speech therapists, neuropsychologists, etc.) who have participated in a specific postgraduate course. This enables one to understand and deepen the theoretical model, receive training in the specific practice, and have supervision for maintaining a high level of methodological fidelity.

Due to its intrinsic characteristics, the SaM method can be applied to patients from the developmental age (from 4 years of age) to adulthood and late adulthood. At present, its effectiveness can still be inferred only through its application in clinical practice. However, the first preliminary data (Risoli et al. 2015) suggested that this approach is flexible, ecological, and effective in rehabilitating complex disabilities.

Activities can be tailored to the specific strengths and weaknesses of each patient, always considering the natural history of the disorder and potential developing areas of the individual. In fact, if, on the one hand, the SaM method requires good language comprehension by the patient, in particular during the multimodal integration activities, to enable her/him to progressively experience and mentally manipulate the different spaces and to improve executive functions, on the other hand, the substantial use of non-verbal communication, particularly during basic activities, allows the adaptation of the method in more complex pathological conditions and also with younger children (< 4 years of age) or with children/adults with very low body awareness or intellectual disabilities. According to this approach, the interaction between the therapist and patient is essentially sensorimotor, with the body serving as the primary means of communication. The therapist’s actions are aimed at facilitating the patient’s voluntary movement toward a specific goal and at motivating her/him through enjoyable strategies that are suitable for their age. For example, the use of errorless learning techniques during basic activities allows the patient to experience the movement of different body districts through immediate positive reinforcement. In this perspective, the therapist guides the patient’s bodily movement and uses prompting strategies to help them experience the correct movement, gradually reducing the prompt to permit voluntary action and enhance awareness of that body part. This may allow the application of some parts of this approach also in patients with limited collaboration, although further studies are necessary to better explore this pathway.

From a multidisciplinary view, the SaM method can play a key role in translating motor and cognitive learning from the clinical environment to the context of daily life. Examples of potential practical applications of the SaM method in children and adults are presented in Table 2.

**Table 2:** Example of possible practical applications of the SaM method

Disorder	
Unilateral Cerebral Palsy (UCP)	Cerebral Palsy is the most common cause of childhood disability, characterised by an impairment in tone, posture and movement (Novak et al. 2017). Although motor symptoms mainly characterise CP, perceptual, cognitive and neuropsychological dysfunctions can also significantly impact CP children’s quality of life. In particular, hemiplegia represents CP’s most common motor manifestation. The European Surveillance of Cerebral Palsy (Kinsner-Ovaskainen et al. 2017) defines hemiplegia as a type of “unilateral cerebral palsy” (UCP) resulting from brain damage related to only one hemisphere, occurring during pre-, peri-, or post-natal period. Specifically, impairment results in the involvement of one body side, opposite to the brain damage. However, atypical neuropsychological development may also be present in this population, mainly represented by deficits in executive functions, visual exploration and attention, and functions related to school learning. Considering the complexity of UCP, early and continuative rehabilitation can include interventions aimed at optimising brain plasticity to promote the child’s motor and cognitive function reorganisation, preventing secondary complications, and improving caregivers’ well-being (Novak et al. 2017). The SaM method may be helpful for this purpose because it can intervene in personal space with activities for the middle axis of the body and for the integration between hemispaces through Basic Activities and Multimodal Integration Activities. Also, in the peripersonal space, Basic Activities and Multimodal Integration Activities may provide easy and complex experiences through the manipulation of different materials (e.g., modelling dough, kinetic sand, etc.) with and without tools, aimed both at developing spatial attention and at encouraging the use of the upper limb for carrying, grasping, with both hands, especially using the properties of the mirror neuron system. Finally, in the extrapersonal space, the SaM Method may focus on both the axes in dynamics and planning abilities through complex activities designed to develop the ability to construct and use mental images, such as performing blindfolded pathways.
Developmental coordination disorder (DCD)	Developmental coordination disorder (DCD) is a neurodevelopmental disorder that significantly impacts an individual’s capacity to acquire and execute fundamental motor tasks essential for daily self-care and well-being (APA 2013). The consequences of DCD encompass difficulties in swiftness and precision in daily life activities that demand motor skills, such as catching, using scissors, handwriting, cycling, and engaging in sports. Furthermore, individuals with DCD may experience lower levels of self-esteem and self-efficacy, along with the manifestation of emotional and behavioural challenges (APA 2013). Children diagnosed with DCD also exhibit poorer academic performance compared to peers, particularly in academic areas such as reading, mathematics, and handwriting. The SaM method operates specifically in what is currently considered in the literature as the specific challenges of DCD (Blank et al. 2019). The method primarily may intervene in personal space with Basic Activities (Rhythmic Movements, Axis in Dynamics, Exploration of Hemispaces) that promote the temporal organisation of movement and multisensory representation of the body axis and hemispaces. Moreover, Multimodal Integration Activities for personal, peripersonal and extrapersonal spaces (e.g., Point Contacts, Manipulations, Sensorimotor Integration starting from the object, Pathways) aim to promote motor programming and generate motor representations and images. The SaM method starts with simple actions and uses affordable materials for DCD children. Successively, through a targeted and progressive program, it will be proposed complex activities, where the executive component of the actions becomes essential (e.g. Phase 3- the activity of Mental Image Rotation and Phase 4 - Mind Maps). This rehabilitation approach provides generalisations process in daily life activities and learning.
Autism Spectrum Disorder (ASD)	Autism Spectrum Disorder (ASD) is a neurodevelopmental disorder with an early insurgence of symptoms that remain present for the entire life of the affected individual. Manifestations of this condition may be very different, and the word “spectrum” means that autism can present very differently in each person (Ozonoff et al. 2014). Although the deficit of reciprocal social communication and the presence of restricted, repetitive, and inflexible patterns of behaviour are the two “core symptoms” (APA 2013), also sensory-motor difficulties are frequent in this population (Bhat et al. 2011; Purpura et al. 2022), and have a persistent impact on daily activities. The motor problems seem to be linked to difficulties in intentionally organising actions. Thus, patients with ASD would not be able to understand the actions of others due to impairments in their motor programming (Fabbri-Destro et al. 2009; Turi et al. 2017). For this reason, sensory-motor aspects should be considered in early and late diagnosis and interventions. Applying the SaM method in ASD individuals may help promote appropriate experience in body space through Basic Activities that may facilitate regulation of posture and intentional action, along with the use of Rhythmic Activities that focus on self and others’ movement. Subsequently, Multimodal Integration Activities, such as Point Contacts, Body Positions or The Manipulation with Tools Leaving Tracks, can focus the rehabilitation program on motor intentionality, action planning and generalisation. Specifically, in Phases 0 and 2 of the intervention, the intersubjective competence, which is possible thanks to a more conscious use of the body, is acted on using the activities of the most suitable method for the subject.
Traumatic brain injury (TBI)	TBI is an alteration of brain functioning and/or structure caused by external mechanical force, where the most frequently affected structures are the orbitofrontal and the anterior temporal cortices and it is the leading cause of death and disability worldwide among trauma-related injuries (Rubiano et al. 2015). Motor and neuropsychological symptoms may be present not only during the acute condition but also in the long term. Commonly impairments are usually observed in language, memory, attention, and executive functions (Brett et al. 2023). Such high-order cognitive abilities are involved in temporal processing, which is crucial in everyday activities and social behaviours, and is also impaired by TBI (Mioni et al. 2014). Action programs and performance, as well as the management of the personal and peripersonal spaces, are usually affected, especially in severe TBI (Falchook et al. 2015). Early and constant rehabilitation can improve the symptomatology, strengthening the progress got in the acute phase. In this context, the SaM method has been shown to be helpful for recovering daily-life adaptive abilities (Risoli et al. 2015). The SaM method addressed to TBI involves different modalities and techniques, according to the phase of recovery of the patients and their characteristics. In the first phase of recovery, Basic Activities focused on the personal space are used to assist the individual in recovering (i) the integrity of the body experienced and (ii) a basic temporal organisation. Subsequently, Multimodal Integration Activities for personal and peripersonal space are introduced to intervene in reorganising action programming and foster cognitive functions, such as working memory, which is fundamental in daily activities. As the patient progresses, the intervention increasingly focuses on executive processes, focusing on activities in peripersonal and especially extrapersonal space, with a clear intention to promote the reorganisation of executive functions to foster the patient’s autonomy.
Unilateral Neglect (UN)	UN usually occurs after a cerebrovascular event and is commonly defined as a disorder involving perception, attention, imagination, and action in the space opposite to the cerebral lesion (generally, in the right hemisphere). Patients affected by UN display slow and/or inaccurate or missing responses in the neglected hemisphere involving personal, peripersonal, and extrapersonal spaces, becoming aware of the affected hemispace or of stimulus occurring in it when they are cued to do so (Vallar and Calzolari 2018). It is also associated with impairment in everyday-life functioning and a slowdown in the recovery of other abilities, such as motor skills and non-spatial attention. Usually, rehabilitation encompasses both top-down and bottom-up techniques, training patients to gaze and make movements towards the affected hemispace (top-down) and manipulating the patients’ sensory environment for instance by vestibular stimulations (bottom-up) (Azouvi et al. 2017). In this context, the SaM Method focuses on promoting spatial attention and mental representation of different spaces through Basic and Multimodal Integration Activities, with activities that require the involvement of both hemispheres simultaneously or alternately. The Basic Activities are also aimed at intervening firstly on personal space; Multimodal Integration Activities in different spaces should always be preceded by targeted Basic Activities (such as rhythmic movements involving the body areas related to the subsequent activity) to foster attentional direction.
Parkinson’s disease (PD)	PD is the second most common neurodegenerative disease, and it mainly results from the loss of dopaminergic neurons in the mesencephalic substantia nigra pars compacta and an accumulation of the protein α-synuclein, which forms Lewy bodies. As the disease progresses, other brain regions are involved, including cortical regions, such as the prefrontal cortex, and the corticostriatal pathways (Bai et al. 2022; Chen et al. 2024). PD is mainly characterized by motor symptoms (such as bradykinesia, rigidity, resting tremor, postural instability, and, mainly in the advanced stages, abnormal postures such as lateral trunk deviations). Also, sensory symptoms and cognitive difficulties can occur, in particular involving attention, executive functions, decision making, visuospatial abilities, and learning (Kawashima et al. 2021). Moreover, evidence shows that PD patients can display impairments in temporal processing, strictly linked with attention, working memory, and movement (Harrington et al. 2011), and in body schema representation and its plasticity, with possible consequences on the interaction with the personal and peripersonal space. The SaM method is specifically focused on the representation of the body schema through all Personal Space Activities. Multimodal Integration Activities intervene in the interaction between the body and the peripersonal space. The application of Multimodal Integration Activities allows to support attention and working memory. As these patients usually present temporal difficulties, the rhythmic temporal aspects of movement appear particularly important in the rehabilitation process based on the Method. For instance, rhythmic movements are employed in various body regions and aim to improve posture and walking.

## Future directions

In recent decades, our understanding of the organisation of motor behaviour and the importance of motor experiences in acquiring or recovering cognitive abilities (such as executive functions) has significantly increased. Data from neuroanatomical, neurophysiological, and behavioural studies in neuroscience can be used to enhance the effectiveness of rehabilitative interventions. In fact, due to congenital or acquired disorders, individuals who display difficulties in egocentric body representations and action planning in space, as well as in action programming and executive functions, can benefit from the SaM method proposed in this paper. The activities of the SaM method may promote motor learning, cognitive flexibility, and motor adaptation in atypical conditions if implemented in an ecological context. Moreover, for its specific focus on embodied cognition and the physical interaction with the surrounding environment through the body, the application of the SaM method, based on more recent data from applied neuroscience, could be used with people who have difficulties in organising voluntary movements, to prepare the adequate cognitive prerequisites necessary to initiate more complex and technological interventions, such as those using virtual reality. Obviously, further studies are required and ongoing, involving single-case observations and larger samples, to support evidence of the Method. In particular, randomised studies are needed to strengthen the generalizability of the findings and better understand the feasibility and efficacy of embodied cognition principles in rehabilitative interventions for different clinical populations.
